# Discrimination of MSA-P and MSA-C by RT-QuIC analysis of olfactory mucosa: the first assessment of assay reproducibility between two specialized laboratories

**DOI:** 10.1186/s13024-021-00491-y

**Published:** 2021-12-11

**Authors:** Connor Bargar, Chiara Maria Giulia De Luca, Grazia Devigili, Antonio Emanuele Elia, Roberto Cilia, Sara Maria Portaleone, Wen Wang, Irene Tramacere, Edoardo Bistaffa, Federico Angelo Cazzaniga, Giovanni Felisati, Giuseppe Legname, Alessio Di Fonzo, Rong Xu, Steven Alexander Gunzler, Giorgio Giaccone, Roberto Eleopra, Shu Guang Chen, Fabio Moda

**Affiliations:** 1 Department of Pathology, Case Western Reserve University School of Medicine, Cleveland, OH USA; 2 Unit of Neurology 5 and Neuropathology, Fondazione IRCCS Istituto Neurologico Carlo Besta, Milan, Italy; 3 Department of Neuroscience, Scuola Internazionale Superiore di Studi Avanzati (SISSA), Trieste, Italy; 4 Unit of Neurology 1 - Parkinson and Movement Disorders, Fondazione IRCCS Istituto Neurologico Carlo Besta, Milan, Italy; 5 Department of Health Science, Santi Paolo e Carlo Hospital and Università degli Studi di Milano, Milan, Italy; 6 Scientific Directorate, Fondazione IRCCS Istituto Neurologico Carlo Besta, Milan, Italy; 7 Unit of Neurology, Foundation IRCCS Ca’ Granda Ospedale Maggiore, Milan, Italy; 8 Department of Population and Quantitative Health Sciences, Case Western Reserve University School of Medicine, Cleveland, OH USA; 9 Department of Neurology, Case Western Reserve University School of Medicine, Cleveland, OH USA; 10 Department of Neurology, University Hospitals Cleveland Medical Center, Cleveland, OH USA

**Keywords:** Alpha-synuclein, Olfactory mucosa, Real-Time Quaking-Induced Conversion, Misfolding, Biomarkers

## Abstract

**Background:**

Detection of the pathological and disease-associated alpha-synuclein (αSyn^D^) in the brain is required to formulate the definitive diagnosis of multiple system atrophy (MSA) and Parkinson’s disease (PD). We recently showed that αSyn^D^ can be detected in the olfactory mucosa (OM) of MSA and PD patients. For this reason, we have performed the first interlaboratory study based on α-synuclein Real-Time Quaking-Induced Conversion (αSyn_RT-QuIC) analysis of OM samples collected from PD and MSA patients with the parkinsonian (MSA-P) and cerebellar (MSA-C) phenotypes.

**Methods:**

OM samples were prospectively collected from patients with a probable diagnosis of MSA-P (*n* = 20, mean disease duration 4.4 years), MSA-C (*n* = 10, mean disease duration 4 years), PD (*n* = 13, mean disease duration 8 years), and healthy control subjects (HS) (*n* = 11). Each sample was analyzed by αSyn_RT-QuIC in two independent specialized laboratories, one located in Italy (ITA-lab) and one located in the USA (USA-lab). Both laboratories have developed and used harmonized αSyn_RT-QuIC analytical procedures. Results were correlated with demographic and clinical data.

**Results:**

The αSyn_RT-QuIC analysis reached a 96% interrater agreement of results (IAR) between laboratories (Kappa = 0.93, 95% CI 0.83–1.00). In particular, αSyn_RT-QuIC seeding activity was found in the OM of 9/13 patients with PD (sensitivity 69%, IAR 100%) and 18/20 patients with MSA-P (sensitivity 90%, IAR 100%). Interestingly, samples collected from patients with MSA-C did not induce αSyn_RT-QuIC seeding activity, except for one subject in USA-lab. Therefore, we found that MSA-P and MSA-C induced opposite effects. Regardless of disease diagnosis, the αSyn_RT-QuIC seeding activity correlated with some clinical parameters, including the rigidity and postural instability.

**Conclusions:**

Our study provides evidence that OM-αSyn^D^ may serve as a novel biomarker for accurate clinical diagnoses of PD, MSA-P, and MSA-C. Moreover, αSyn_RT-QuIC represents a reliable assay that can distinguish patients with MSA-P from those with MSA-C, and may lead to significant advancements in patients stratification and selection for emerging pharmacological treatments and clinical trials.

**Supplementary Information:**

The online version contains supplementary material available at 10.1186/s13024-021-00491-y.

## Background

Multiple system atrophy (MSA) is a rare and rapidly progressing neurodegenerative disorder that can be classified into at least two main subtypes: the parkinsonian (MSA-P) and the cerebellar (MSA-C) types [1]. MSA-P shows prominent parkinsonism, such as tremors and postural instability, while MSA-C shows cerebellar ataxia [2, 3]. Diagnosis of MSA is challenging because the clinical presentation often overlaps with that of other α-synucleinopathies, including Parkinson’s disease (PD), especially at the early stages. Nonetheless, MSA patients may display cruciform hypo-intensity in the pons (hot cross bun) and hyperintensity in the dorsal margin of the putamen (putaminal slit) by magnetic resonance imaging (MRI), as well as normal cardiac uptake of I-123 MIBG and normal olfactory function. In contrast, these two latter aspects are often impaired in PD patients [4–6]. Although these tests allow clinical diagnosis of MSA, about 20 to 40% of patients are still misdiagnosed, therefore better disease biomarkers are urgently needed [7].

A definitive diagnosis of α-synucleinopathies relies on the detection in the brain of protein aggregates made of misfolded and disease-associated alpha-synuclein (αSyn^D^) [8, 9]. Notably, the neuroanatomical distribution and morphological and biochemical features of αSyn^D^ aggregates differ between MSA and PD, and the reasons for this variability seem to be enciphered in the structure of αSyn^D^ that gives rise to distinct strains [7, 10, 11]. Thanks to the advent of the Real-Time Quaking-Induced Conversion (RT-QuIC) assay, traces of αSyn^D^ were detectable in cerebrospinal fluid (CSF), olfactory mucosa (OM), and skin of patients with MSA, PD, and other α-synucleinopathies, with high sensitivity and specificity [12–19]. Hence, the RT-QuIC has been increasingly used to assist in the premortem diagnosis of these pathologies, especially with regard to CSF analysis. However, the current lack of harmonized analytical procedures between laboratories hampers the possibility to carefully evaluate the overall reliability and robustness of the assay in clinical practice.

At present, only one CSF-based RT-QuIC comparative study for the diagnosis of PD has been reported [20].

An international cross-laboratory validation study of OM-based RT-QuIC analysis has never been performed and may help to carefully evaluate the sensitivity and specificity of the assay toward the diagnosis of PD, MSA-P, and MSA-C using this biological sample. To this aim, we combined the specific RT-QuIC expertise of two laboratories, one in Italy (ITA-lab) and one in the USA (USA-lab), to set up a precise α-synuclein RT-QuIC (αSyn_RT-QuIC) protocol for the analysis of OM samples collected from well-characterized patients with MSA-P, MSA-C, PD, and healthy subjects (HS). After extensive technical optimizations, we established a protocol that fulfilled our analytical needs and yielded a very high interlaboratory reproducibility of the test results. To the best of our knowledge, this is the first interlaboratory study of OM samples and our results indicate that αSyn_RT-QuIC may be a robust and reliable biochemical assay to accurately diagnose and discriminate PD, MSA-P, and MSA-C in living patients using an easily collectible tissue. The identification of peripheral αSyn^D^ will significantly increase the accuracy of premortem diagnosis and facilitate patients’ stratification for therapeutic treatments and enrollment in clinical trials.

## Methods

### Study design and participants

This exploratory and prospective study involved two different laboratories, one at the Fondazione IRCCS Istituto Neurologico Carlo Besta in Italy (ITA-lab) and the other one at the Case Western Reserve University School of Medicine in USA (USA-lab). A consecutive series of well-characterized patients followed at the Parkinson and Movement Disorders Unit of ITA-lab who met established diagnostic criteria for probable MSA-P (*n* = 20, 55% male, mean age 60 years, mean disease duration 4.4 years), MSA-C (*n* = 10, 60% male, mean age 61 years, mean disease duration 4 years), PD (*n* = 13, 62% male, mean age 63 years, mean disease duration 8 years), and a group of HS (*n* = 11, 45% male, mean age 42 years) were included in the study (Table 1) [1, 21]. The age differences between patients and healthy subjects (61 ± 8 and 42 ± 10 years, respectively) as well as the disease duration between MSA and PD patients (4 ± 3.1 and 8 ± 4.3 years, respectively) were statistically significant (t-test *p* < 0.01). Extensive clinical symptoms and signs, including treatment response, presence of REM sleep behavior disorder, and red flags helping the MSA diagnosis were carefully assessed and detailed in Tables 2 and 3. The clinical diagnosis was confirmed by three independent clinicians (GD, AEE, RE) and supported by specific diagnostic tests including brain MRI, cardiovascular autonomic tests, nigrostriatal dopamine transporter imaging with 123I-ioflupane single-photon emission computed tomography, and cardiac uptake of I-123 MIBG single-photon emission computed tomography. Motor symptoms were assessed using part III of the Movement Disorder Society-Unified Parkinson’s Disease Rating Scale (MDS-UPDRS) [22]. Patients suspected to have other neurodegenerative diseases (e.g. Alzheimer’s disease, progressive supranuclear palsy, corticobasal syndrome, and dementia with Lewy bodies) or other relevant clinical conditions (including stroke, neuromuscular diseases, severe osteoarthrosis, or other musculoskeletal impairments affecting gait and standing) that could have affected the clinical scales (e.g. MDS-UPDRS III, H&Y) were excluded from the study. Similarly, patients unable to provide their informed consent were not included. All the procedures involving human participants were performed in accordance with the ethical standards of the institutional and/or national research committees and with the 1964 Helsinki declaration. The study and its ethical aspects were approved by the Fondazione IRCCS Istituto Neurologico Carlo Besta ethical committee. All of the participants provided written informed consent before OM collection and analysis.

**Table 1:** Demographic data and αSyn_RT-QuIC results

Clinical diagnosis	Sex (Male)	Year of birth	Age at onset	Age at brushing	T1 (years) = disease duration	Clinical onset	Family history of parkinsonism or ataxia	Time to threshold		Fluorescence at time threshold		Mean intensity at PK 2.5 mg/mL (ITA-lab)	αSyn_RT-QuIC results	
								ITA-lab	USA-lab	ITA-lab	USA-lab		ITA-lab	USA-lab
PD_1	+	1956	55	61	6	(rest) tremor at left hand	–	9.00	9.44	260,000	260,000	9.56	+	+
PD_2	–	1947	63	71	8	rigidity right hand	–	9.5	11.25	260,000	250,509	5.67	+	+
PD_3	+	1952	54	66	12	bradykinesia and rigidity left hand	–	10.25	12.44	171,531	260,000	5.59	+	+
PD_4	–	1959	50	59	9	rigidity right hand	–	NA	NA	14,452	6607	5.85	–	–
PD_5	+	1945	61	74	13	rigidity and tremor	–	NA	NA	14,026	7283	7.02	–	–
PD_6	+	1967	48	52	4	dysautonomia	+	NA	NA	13,370	13,623	6.41	–	–
PD_7	+	1967	49	51	2	bradykinesia and tremor	–	7.5	14.13	259,150	260,000	59.39	+	+
PD_8	–	1952	50	67	17	bradykinesia and rigidity left hand	–	NA	NA	17,385	8232	5.29	–	–
PD_9	–	1950	59	67	8	rigidity	–	11.33	19.5	121,284	49,958	12.32	+	+
PD_10	+	1961	53	58	5	dysautonomia	–	10.33	21.00	120,929	55,342	6.63	+	+
PD_11	+	1962	52	58	6	rigidity and frozen shoulder	–	8.88	15.08	169,629	159,598	8.19	+	+
PD_12	–	1947	69	72	3	bradykinesia and rigidity left leg	+	9.00	20.25	222,667	49,532	6.49	+	+
PD_13	+	1951	55	66	11	bradykinesia and tremor	–	8.00	20.25	260,000	35,814	11.58	+	+
MSA-P_1	–	1947	67	70	3	dysautonomia	–	10.67	14.56	183,784	215,981	81.11	+	+
MSA-P_2	+	1948	69	71	2	parkinsonism	–	10.25	20.88	173,619	56,919	56.09	+	+
MSA-P_3	+	1964	48	53	5	RBD and later parkinsonism	–	10.00	12.69	227,396	239,598	21.67	+	+
MSA-P_4	+	1965	50	53	3	parkinsonism	–	12.33	18.13	41,279	196,898	56.97	+	+
MSA-P_5	+	1957	56	62	6	dysautonomia	–	11.75	17.25	56,628	183,834	21.57	+	+
MSA-P_6	–	1957	54	61	7	parkinsonism	–	10.00	17.08	204,429	206,638	6.71	+	+
MSA-P_7	–	1946	72	73	1	dysautonomia	–	11.50	10.19	86,628	88,018	82.57	+	+
MSA-P_8	+	1973	40	46	6	parkinsonism	–	11.50	16.92	109,166	47,076	9.69	+	+
MSA-P_9	+	1976	39	44	5	dysautonomia	–	8.50	14.50	248,346	59,610	18.36	+	+
MSA-P_10	+	1962	55	57	2	RBD and later parkinsonism	–	NA	NA	NA	NA	7.27	–	–
MSA-P_11	–	1959	58	60	2	parkinsonism	–	NA	NA	NA	NA	6.98	–	–
MSA-P_12	–	1958	57	60	3	parkinsonism	–	9.67	20.75	147,488	135,777	42.32	+	+
MSA-P_13	+	1963	51	56	5	dysautonomia	–	10.50	18.67	218,123	82,606	10.26	+	+
MSA-P_14	–	1963	55	56	1	parkinsonism	–	10.50	17.63	160,382	260,000	18.2	+	+
MSA-P_15	–	1965	41	54	13	parkinsonism	–	9.50	20.33	213,766	84,203	5.77	+	+
MSA-P_16	+	1953	60	64	4	bradykynesia parkinsonism	–	11.00	22.50	133,273	148,704	8.24	+	+
MSA-P_17	+	1949	69	70	1	parkinsonism	–	10.63	19.13	224,756	235,573	95.5	+	+
MSA-P_18	–	1963	61	65	4	parkinsonism	–	8.33	20.00	260,000	30,995	42.27	+	+
MSA-P_19	+	1959	58	60	2	parkinsonism	–	10.33	16.33	183,011	50,658	34.47	+	+
MSA-P_20	–	1953	52	65	13	parkinsonism	+	12.75	12.63	43,645	57,486	5.85	+	+
MSA-C_1	–	1965	45	54	9	cerebellar	–	NA	NA	NA	NA	2.86	–	–
MSA-C_2	+	1947	70	72	2	cerebellar	–	NA	NA	NA	NA	3.07	–	–
MSA-C_3	–	1957	57	62	5	RBD	–	NA	NA	NA	NA	3.09	–	–
MSA-C_4	+	1971	47	48	1	cerebellar	–	NA	NA	NA	NA	3.37	–	–
MSA-C_5	–	1950	64	69	5	cerebellar	–	NA	NA	NA	NA	3.76	–	–
MSA-C_6	+	1964	50	55	5	cerebellar	–	NA	NA	NA	NA	3.86	–	–
MSA-C_7	+	1950	69	70	1	cerebellar	–	NA	NA	NA	NA	4.4	–	+
MSA-C_8	+	1965	51	55	4	dysautonomia	–	NA	NA	NA	NA	4.86	–	–
MSA-C_9	–	1948	67	70	3	cerebellar	–	NA	NA	NA	NA	4.68	–	–
MSA-C_10	+	1965	50	55	5	cerebellar	–	NA	NA	NA	NA	3.59	–	–
HS_1	+	1955	NA	64	NA	NA	NA	NA	NA	NA	NA	4.22	–	–
HS_2	+	1981	NA	38	NA	NA	NA	NA	NA	NA	NA	3.98	–	–
HS_3	–	1985	NA	35	NA	NA	NA	NA	NA	NA	NA	3.03	–	–
HS_4	–	1987	NA	32	NA	NA	NA	NA	NA	NA	NA	3.44	–	–
HS_5	–	1969	NA	50	NA	NA	NA	NA	NA	NA	NA	2.12	–	–
HS_6	+	1979	NA	38	NA	NA	NA	NA	NA	NA	NA	3.33	–	–
HS_7	–	1984	NA	35	NA	NA	NA	NA	NA	NA	NA	3.17	–	–
HS_8	–	1979	NA	39	NA	NA	NA	NA	NA	NA	NA	3.04	–	+
HS_9	+	1974	NA	45	NA	NA	NA	NA	NA	NA	NA6	2.87	–	–
HS_10	–	1970	NA	49	NA	NA	NA	NA	NA	NA	NA	2.21	–	–
HS_11	+	1985	NA	34	NA	NA	NA	NA	NA	NA	NA	2.78	–	–

**Table 2:** Clinical features of the enrolled patients

Clinical diagnosis	Bradykinesia	Rigidity	Tremor	Postural instability	Gait freezing	RBD	Early gait ataxia (3 years of onset)	Gait ataxia	Limb ataxia	Cerebellar Dysartria	Cerebellar Oculomotor disfunction	Hyposmia	Autonomic failure	Orthostatic Hypotension	Other cardiovascular dysautonomic symptoms	Urinary disfunction	Other dysautonomia
PD_1	+	+	+	–	–	–	–	–	–	–	–	–	–	–	–	–	–
PD_2	+	+	+	–	–	+	–	–	–	–	–	–	–	–	+	+	+
PD_3	–	–	–	–	–	–	–	–	–	–	–	–	–	–	–	+	–
PD_4	–	–	–	–	–	–	–	–	–	–	–	–	+	+	+	–	–
PD_5	–	–	–	–	–	–	–	–	–	–	–	–	–	–	–	+	+
PD_6	+	+	+	–	+	+	–	–	–	–	–	+	+	+	+	+	–
PD_7	+	+	+	–	–	–	–	–	–	–	–	+	–	–	–	–	+
PD_8	+	+	–	–	–	–	–	–	–	–	–	–	–	–	–	–	–
PD_9	+	+	–	–	–	–	–	–	–	–	–	+	–	–	–	–	–
PD_10	+	+	–	+	–	–	–	–	–	–	–	–	+	+	+	+	+
PD_11	+	+	+	–	–	–	–	–	–	–	–	+	+	–	–	+	–
PD_12	+	+	–	–	–	+	–	–	–	–	–	–	–	–	–	–	–
PD_13	+	+	+	–	+	–	–	–	–	–	–	–	+	–	–	+	+
MSA-P_1	+	+	+	+	–	–	+	+	+	+	–	–	+	+	+	+	+
MSA-P_2	+	+	+	+	–	–	–	–	–	–	–	–	+	–	+	+	+
MSA-P_3	+	+	+	+	–	+	–	–	–	–	–	–	+	+	+	+	+
MSA-P_4	+	+	+	+	+	+	–	–	–	–	–	–	+	+	+	+	+
MSA-P_5	+	+	–	+	–	+	–	+	+	+	–	–	+	+	+	+	+
MSA-P_6	+	+	+	+	–	+	–	+	+	+	–	–	+	+	+	+	+
MSA-P_7	+	+	–	+	–	–	–	+	+	+	–	–	+	+	+	+	+
MSA-P_8	+	+	+	+	–	–	–	–	–	–	–	–	+	+	+	+	+
MSA-P_9	+	+	+	+	–	+	–	–	–	–	**–**	–	+	+	+	+	+
MSA-P_10	+	+	+	–	–	+	–	–	–	–	**–**	–	+	+	+	+	+
MSA-P_11	+	–	–	+	+	–	–	+	–	–	**–**	–	+	+	+	+	+
MSA-P_12	+	+	+	+	–	+	–	–	–	–	**–**	–	+	–	+	+	+
MSA-P_13	+	+	+	+	–	+	–	–	–	–	**–**	–	+	+	+	+	+
MSA-P_14	+	+	+	+	–	+	–	–	–	–	**–**	+	+	+	+	+	+
MSA-P_15	+	+	+	+	–	+	–	–	–	–	**–**	–	+	–	+	+	+
MSA-P_16	+	+	+	+	–	–	–	–	–	–	**–**	–	+	+	+	+	+
MSA-P_17	+	+	+	+	–	–	–	–	–	–	**–**	–	+	+	+	+	+
MSA-P_18	+	+	–	+	–	–	–	–	–	+	**–**	–	+	–	+	+	+ (sudomotor)
MSA-P_19	+	+	–	+	–	–	–	–	–	–	**–**	–	+	+	+	+	+
MSA-P_20	+	+	–	+	–	–	–	+	+	+	**–**	–	+	–	+	+	+
MSA-C_1	+	+	–	+	–	+	+	+	+	+	**–**	–	+	+	+	+	+ (stipsi)
MSA-C_2	–	–	–	–	–	–	+	+	+	+	**–**	+	+	–	+	–	–
MSA-C_3	+	–	–	–	–	+	+	+	+	+	**+**	–	+	–	+	+	+ (stipsi)
MSA-C_4	–	+	–	+	–	–	+	+	+	+	**+**	+	+	–	+	+	+ (stipsi)
MSA-C_5	+	–	–	+	–	+	+	+	+	+	**+**	–	+	–	+	+	+ (sudomotor)
MSA-C_6	+	+	–	+	–	–	+	+	+	+	**+**	–	+	–	+	+	+ (genital)
MSA-C_7	–	–	–	+	–	+	+	+	+	+	**+**	–	+	–	+	–	–
MSA-C_8	+	–	–	+	–	+	+	+	+	+	**+**	–	+	+	+	+	+
MSA-C_9	–	–	–	+	–	–	+	+	+	+	**+**	–	–	–	–	–	+
MSA-C_10	+	–	–	+	–	+	+	+	+	+	**+**	–	+	+	+	+	+

**Table 3:** Instrumental findings and additional features supportive for the clinical diagnosis

Clinical diagnosis	Disease duration at sampling (years)	Parkinsonism	Beneficial L-Dopa response (MDS-UPDRS-III CHANGE %)	Cerebellar syndrome	MRI abnormalities			FDG-PET	SPET-DATSCAN	MIBG
					Putaminal slit sign	Middle cerebellar peduncle	Cerebellum atrophy			
PD_1	6	+	37	–	–	–	–	NA	+	NA
PD_2	8	+	48	–	–	–	–	NA	+	NA
PD_3	12	+	27	–	–	–	–	NA	+	NA
PD_4	9	+	29	–	–	–	–	NA	+	+
PD_5	13	+	31	–	–	–	–	NA	+	NA
PD_6	4	+	23	–	–	–	–	NA	+	+
PD_7	2	+	27	–	–	–	–	NA	+	NA
PD_8	17	+	31	–	–	–	–	NA	+	NA
PD_9	8	+	29	–	–	–	–	NA	+	NA
PD_10	5	+	30	–	–	–	–	NA	+	+
PD_11	6	+	21	–	–	–	–	-	+	+
PD_12	3	+	35	–	–	–	–	–	+	+
PD_13	11	+	23	–	–	–	–	–	+	NA
MSA-P_1	3	+	29	+	+	+	+	NA	+	–
MSA-P_2	2	+	14	–	+	–	–	NA	+	–
MSA-P_3	5	+	24	–	+	–	–	NA	+	NA
MSA-P_4	3	+	16	–	+	+	+	NA	+	–
MSA-P_5	6	+	18	+	–	–	–	NA	+	–
MSA-P_6	7	+	12	–	–	+	+	NA	+	NA
MSA-P_7	1	+	12	+	+	–	–	NA	+	+
MSA-P_8	6	+	16	–	+	–	–	NA	+	–
MSA-P_9	5	+	9	–	+	–	–	NA	+	mild pos
MSA-P_10	2	+	6	–	+	–	+	NA	+	-
MSA-P_11	2	+	10	+	+	–	+	NA	+	-
MSA-P_12	3	+	13	–	+	–	+	NA	+	–
MSA-P_13	5	+	22	–	–	–	–	NA	+	-
MSA-P_14	1	+	13	–	–	–	–	NA	+	–
MSA-P_15	13	+	14	–	–	–	–	-	+	–
MSA-P_16	4	+	9	–	–	–	–	-	+	-
MSA-P_17	1	+	11	–	–	–	–	-	–	-
MSA-P_18	4	+	0	+	–	–	–	-	–	+
MSA-P_19	2	+	5	–	–	–	–	-	–	-
MSA-P_20	13	+	13	+	+	+	+	-	+	NA
MSA-C_1	9	+	14	+	–	–	–	NA	+	NA
MSA-C_2	2	–	10	+	+	+	+	NA	**+**	–
MSA-C_3	5	–	22	+	+	+	+	NA	+	NA
MSA-C_4	1	–	13	+	+	+	+	NA	**+**	–
MSA-C_5	5	+	14	+	+	+	+	NA	**+**	–
MSA-C_6	5	+	31	+	+	+	+	NA	**+**	NA
MSA-C_7	1	–	29	+	+	+	+	+	**-**	NA
MSA-C_8	4	–	22	+	–	+	+	+	**-**	–
MSA-C_9	3	–	11	+	–	+	+	NA	**-**	NA
MSA-C_10	5	+	10	+	+	–	–	NA	**+**	–

### Collection of OM samples

OM samples were collected by a specialized otorhinolaryngologist with non-invasive procedures, as previously reported [16], and before the COVID-19 pandemic. A total of 54 OM samples were collected by gently brushing between the septum and the middle turbinate in the nasal vault with a flocked swab (FLOQSwabs™ Copan Italia, Brescia, Italy). All OM samples were collected at ITA-lab, processed, and divided into two aliquots: one for ITA-lab and the other for USA-lab analyses. Samples were blindly tested by both laboratories.

### Preparation of OM for αSyn_RT-QuIC analysis

OM samples were prepared as previously described [16]. Six μg of pelleted cells was suspended in 50 μL of phosphate-buffer saline (PBS), while the remaining pellet of OM was stored at − 80 °C. Ten microliters of the sample was then diluted 20 folds in PBS (supplemented with 1% Triton-X 100) and the resulting solution was analyzed by αSyn_RT-QuIC.

### Harmonized protocol for αSyn_RT-QuIC analysis

The αSyn_RT-QuIC protocol for OM samples analysis was modified from that of a previous report [16] with the consideration of salt effects on RT-QuIC reactivity as recently described [23]. In brief, αSyn_RT-QuIC analyses were performed using 384-well optical flat bottom plates (Thermo Scientific). Each well was preloaded with two low-binding silica beads (0.8 mm, OPS Diagnostics). Lyophilized recombinant human αSyn (rec-αSyn), purchased from rPeptide (S-1001-2), was reconstituted in water and filtered with 100 kDa filters immediately before use. For each well, 1 μL of OM samples was added to 49 μL of αSyn_RT-QuIC reaction mix that was composed of: rec-αSyn [0.1 mg/mL], HEPES (pH 8.0) [40 mM], sodium citrate [170 mM], and thioflavin-T (ThT) [20 μM]. Each sample was analyzed in quadruplicate and at least three times in both labs. The plates were incubated at 42 °C in a BMG FluoSTAR OMEGA microplate reader (BMG Labtech) and subjected to cycles of shaking and incubation (1 min each). Under this condition, rec-αSyn undergoes self-assembly and forms amyloid fibrils following well-defined kinetics. The addition of a biological sample (e.g. OM), containing minute amounts of αSyn^D^, significantly increases this aggregation kinetics, producing the so-called *seeding activity*. Fluorescence intensities of ThT, expressed as arbitrary units (AU), were taken every 30 min using 450 ± 10 nm (excitation) and 480 ± 10 nm (emission) wave-lengths, with a bottom read. A sample was considered able to induce seeding activity when at least two out of four replicates crossed the threshold of fluorescence set at 30,000 AU before 13 h at ITA-lab or 22.5 h at USA-lab. In this case, the sample was considered positive (+). For each positive sample, we have calculated the average fluorescence intensity of the replicates that crossed the fluorescence thresholds and plotted the results in a graph against time. Results were correlated with demographic and clinical data (Table 5).

#### Dot blot analysis of αSyn_RT-QuIC reaction products

Two μL of final αSyn_RT-QuIC products was diluted with 500 μL of PBS containing 0.1% Tween-20. One hundred microliters of the diluted samples were loaded onto a nitrocellulose membrane (0.2 μm pore) assembled in a Bio-Rad 96-well Bio-Dot apparatus per manufacturer’s protocol. The membrane was blocked for 1 h at room temperature with 5% bovine serum albumin (BSA) made in PBS (5% BSA-PBS). The membrane was then incubated for 1 h with a rabbit monoclonal αSyn filament-specific antibody (Abcam ab209538, diluted 1:5,000 in 5% BSA-PBS) or a rabbit polyclonal total αSyn antibody (Millipore Ab5038, diluted 1:1,000 in 5% BSA-PBS). After washing 3 times with Tris-buffered saline containing 0.1% Tween-20 (TBST-0.1), the membrane was incubated for 1 h with a secondary antibody (Amersham donkey against rabbit IgG-HRP, diluted 1:10,000 in 5% BSA-PBS). Following three washes with TBST-0.1, the immunoreactivity on the dot-blotted membrane was developed with the enhanced chemiluminescence reagents (ECL, Amersham). The densitometric quantitation of the individual dots was performed with the Epson Perfection V600 photo scanner (Epson Scan Utility v3.9.2).

#### Protease resistance analysis of αSyn_RT-QuIC reaction products

Eight μL of final αSyn_RT-QuIC products was treated with proteinase K (PK, Invitrogen) [2.5 mg/mL] for 1 h at 37 °C (under shaking at 500 rpm). PK digestions were stopped by the addition of the LDS-PAGE loading buffer (Bolt™ LDS Sample Buffer and DTT, Thermo Scientific) and by boiling the samples at 100 °C for 10 min (under shaking at 500 rpm). Proteins were then separated by means of SDS-PAGE, using 12% Bis-Tris plus gels (Thermo Scientific), and transferred onto polyvinylidene difluoride (PVDF) membranes (Immobilon-P, Millipore). PVDF membranes were incubated with paraformaldehyde (0.4% in PBS) for 30 min at room temperature (under shaking) and subsequently incubated with non-fat dry milk (prepared in TBS with 0.05% Tween-20, TBST-0.05) for 1 h at room temperature. Membranes were then incubated with a rabbit polyclonal α-synuclein antibody (Agrisera AS08 358, diluted 1:1000 in TBST-0.05) overnight at 4 °C (under shaking). After three washes in TBST-0.05, membranes were incubated with a secondary antibody (Amersham donkey against rabbit IgG-HRP, diluted 1:2,000 in TBST-0.05 supplemented with 5% non-fat dry milk) for 1 h at room temperature (under shaking) and developed with a chemiluminescent system (ECL Prime, Amersham). Finally, reactions were visualized using a G:BOX Chemi Syngene system. Densitometric analysis of Western blot bands was carried out using ImageJ software (1.51v).

### Statistical analysis

αSyn_RT-QuIC kinetics were represented using the Prism software (GraphPad v9.0.0) on a graph where mean fluorescence values were plotted against time. For each patient, we calculated the average times at which the positive replicates crossed the predefined threshold of 30,000 AU (time to threshold). The mean fluorescence intensity reached by each patient at 13 h at ITA-lab or 22.5 h at USA-lab was used to calculate the fluorescence values at the time threshold. Graphic representations of densitometric analysis were performed using the Prism software (GraphPad v9.0.0). Associations between variables were investigated through t-test or Mann-Whitney test, Chi-square or Fisher exact test, as appropriate. Dot blot results and PK resistance profiles of αSyn_RT-QuIC products generated by PD and MSA were analyzed through repeated measure analysis of variance (ANOVA). Given the exploratory nature of our study, the calculation of the power analysis was not performed. Kappa statistic with the corresponding 95% confidence interval (CI) was calculated to assess interrater agreement between ITA-lab and USA-lab.

## Results

### Efficient αSyn_RT-QuIC seeding activity was observed in OM of PD and MSA-P patients but not in those of MSA-C patients

The USA-lab introduced some important modifications to the RT-QuIC protocol published in 2019 [16] by some of the authors of this paper for the analysis of OM samples.

Following the validation of the αSyn_RT-QuIC protocol using the brain homogenates of autopsy-confirmed cases of PD, MSA-P, and MSA-C as sources of different αSyn^D^ strains (see Supplementary Fig. 1), ITA-lab and USA-lab performed blind αSyn_RT-QuIC analyses of OM samples collected from PD (% mean MDS-UPDRS Gain 30.1), MSA-P (% mean MDS-UPDRS Gain 13.3), MSA-C (% mean MDS-UPDRS Gain 17.6) patients as well as HS. The demographic data and the αSyn_RT-QuIC results, the clinical features and the instrumental findings of the patients included in the study are reported in Tables 1, 2 and 3, respectively.

**Fig. 1:**
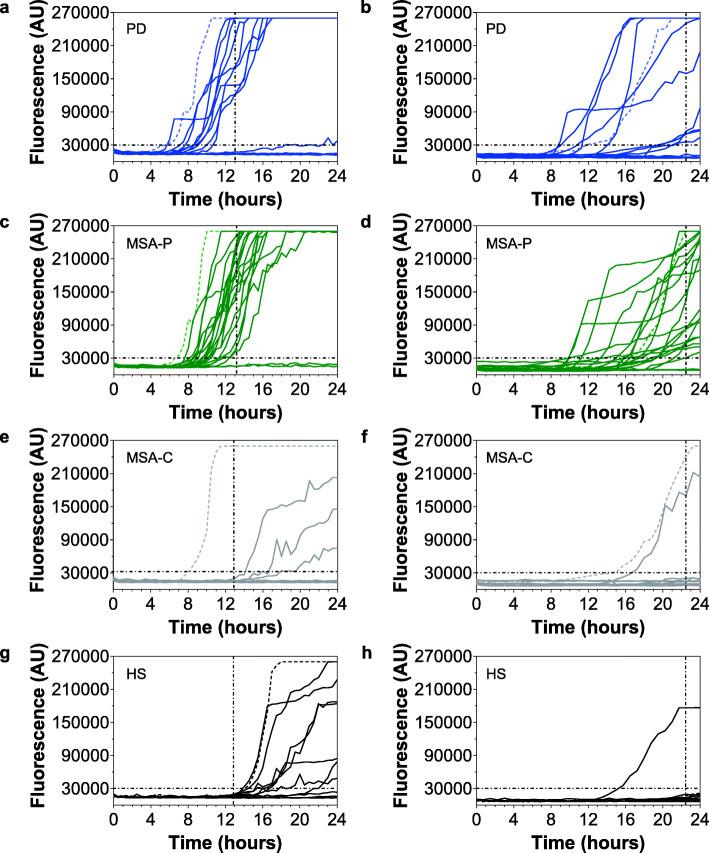
Evaluation of αSyn_RT-QuIC seeding activity triggered by the OM of patients with PD, MSA-P, MSA-C, and HS. RT-QuIC seeding activity of αSyn^D^ was observed in 9/13 OM samples of the same PD patients (**a** and **b**) and 18/20 of the same MSA-P patients (**c** and **d**) analyzed at ITA-lab (**a** and **c**) and USA-lab (**b** and **d**). None of the samples collected from MSA-C (**e**) or HS (**g**) induced a seeding activity at ITA-lab, while 1/10 OM sample collected from MSA-C patients (**f**) and 1/11 sample collected from HS (**h**) induced a seeding activity at USA-lab. Samples were analyzed at least three times in quadruplicate. Curves represented in the graphs were obtained by plotting the average fluorescence intensities of each sample against time. The brain homogenates (BH) of PD (dashed curves in **a** and **b**), MSA-P (dashed curves in **c** and **d**) and MSA-C (dashed curves in **e** and **f**) patients were diluted until 10^− 4^ and used as positive controls. The BH of a patient not affected by α-synucleinopathy (dashed curves in **g** and **h**) was diluted until 10^− 4^ and used as negative control

Graphical representations of the αSyn_RT-QuIC seeding activities of each OM sample obtained in both labs are shown in Fig. 1. According to lab-specific time thresholds, we observed that the OM of the same PD patients (9/13) and MSA-P patients (18/20) consistently induced a seeding activity for rec-αSyn thus yielding a 100% interlaboratory reproducibility of the results (Fig. 1a,b,c,d).

Notably, at ITA-lab the OM of PD patients induced faster rec-αSyn aggregation compared to those of MSA-P and the time to threshold (mean ± standard deviation) of PD (9.31 ± 1.19 h) was statistically different from that of MSA-P (10.54 ± 1.17 h) (t-test, *p* = 0.0170) (Supplementary Fig. 2). In contrast, at USA-lab the time to threshold of PD and MSA-P samples (15.93 ± 4.41 h and 17.23 ± 3.29 h, respectively) was not statistically different (t-test, *p* = 0.3944). Similarly, both laboratories did not observe differences in the average fluorescence values reached by PD and MSA-P at the time thresholds (Supplementary Fig. 2).

With respect to the clinical diagnosis, we obtained a 90% sensitivity of αSyn_RT-QuIC seeding activity in OM samples of patients with MSA-P while that of PD patients was 69% (slightly higher than that published in 2019 [16]) (Table 4). Surprisingly, except for one OM at USA-lab, none of the MSA-C samples induced αSyn_RT-QuIC seeding activity, leading to a 90% interrater agreement of results (IAR) (Fig. 1e,f and Table 4). Thus, the opposite αSyn_RT-QuIC analytical responsiveness of MSA-C and MSA-P, constantly observed in both laboratories, enabled efficient discrimination between disease phenotypes. Finally, none of the OM collected from HS induced seeding activity, except for one sample at USA-lab (Fig. 1g,h and Table 4). This yielded a 91% IAR between laboratories. Taken together, these data led to an overall 96% IAR between ITA-lab and USA-lab (Kappa = 0.93, 95% CI 0.83–1.00) (Table 4). Thus, the optimized αSyn_RT-QuIC analyses enabled high levels of discrimination between (i) MSA-P and MSA-C (chi-square test, *p* < 0.001 based on both ITA-lab and USA-lab findings), (ii) MSA-P and HS (chi-square test, *p* < 0.001 based on both ITA-lab and USA-lab findings), and (iii) PD and HS (chi-square test, *p* ≤ 0.001 and *p* = 0.003 based on ITA-lab and USA-lab findings, respectively) with a specificity of 91%. Notably, repeated cycles of freezing and thawing of the OM samples did not affect their αSyn_RT-QuIC behavior.

**Table 4:** Comparison of αSyn_RT-QuIC results between ITA-lab and USA-lab

	PD		MSA-P		MSA-C		HS	
	ITA-lab	USA-lab	ITA-lab	USA-lab	ITA-lab	USA-lab	ITA-lab	USA-lab
Samples analyzed	13	13	20	20	10	10	11	11
αSyn^D^ seeding activity	9	9	18	18	0	1	0	1
Mean fluorescence at the time threshold % (SD)	56.46 (39.88)	41.85 (42.41)	56.70 (30.76)	46.10 (32.20)	5.90 (0.99)	11.00 (19.40)	6.18 (1.07)	10.00 (19.29)
Mean time at fluorescence threshold (SD)	9.31 (1.19)	15.93 (4.41)	10.54 (1.17)	17.23 (3.29)	NA	NA	NA	NA
Seeding activity vs clinical diagnosis (sensitivity)	69%	69%	90%	90%	0%	10%	NA	NA
Disease-specific interrater agreement between labs	100%		100%		90%		91%	
Overall interrater agreement between labs	**96% (Kappa = 0.93, 95% CI 0.83–1.00)**							

### αSyn_RT-QuIC seeding activity of OM samples significantly correlated with patient rigidity and postural instability

We then performed association analyses between αSyn_RT-QuIC seeding activity in PD and MSA-P patients and several clinical parameters (see Table 5) A significant positive association was found between αSyn_RT-QuIC seeding activity and rigidity (seeding activity was present in 89.7% or 25.0% of patients with vs without rigidity, Fisher exact test, *p* = 0.014) and postural instability (seeding activity was present in 95.0% or 61.5% of patients with vs without postural instability, Fisher exact test, *p* = 0.025). Furthermore, there was a trend toward greater seeding activity prevalence, albeit not statistically significant, in patients with bradykinesia compared to those without symptom (86.7% vs 33.3%, Fisher exact test, *p* = 0.078). Finally, an inverse, although not statistically significant, association was observed between seeding activity and disease duration (7.8 vs 5.4 years in patients without vs with seeding activity respectively, t-test, *p* = 0.19). Similar results were observed considering separately PD and MSA-P patients groups.

**Table 5:** Association analyses between αSyn_RT-QuIC seeding activity triggered by OM of PD and MSA-P patients and clinical or demographic parameters

Clinical/demographic parameter	αSyn_RT-QuIC seeding activity		***p***-value*
	Positive	Negative	
Sex – n (%)			1
Male	16 (84.21)	3 (15.79)	
Female	11 (78.57)	3 (21.43)	
Cerebellar syndrome – n (%)			1
Yes	5 (83.33)	1 (16.67)	
No	22 (81.48)	5 (18.52)	
MRI putamen strie – n (%)			1
Yes	9 (81.82)	2 (18.18)	
No	18 (81.82)	4 (18.18)	
MRI middle cerebellar peduncle – n (%)			1
Yes	4 (100)	0 (0)	
No	23 (79.31)	6 (20.69)	
MRI cerebellum – n (%)			0.58
Yes	5 (71.43)	2 (28.57)	
No	22 (84.62)	4 (15.38)	
Family history of parkinsonisms – n (%)			1
Yes	2 (66.67)	1 (33.33)	
No	25 (83.33)	5 (16.67)	
Bradykinesia – n (%)			0.08
Yes	26 (86.67)	4 (13.33)	
No	1 (33.33)	2 (66.67)	
Rigidity – n (%)			0.01
Yes	26 (89.66)	3 (10.24)	
No	1 (25.00)	3 (75.00)	
Tremor – n (%)			0.18
Yes	18 (90.00)	2 (10.00)	
No	9 (69.23)	4 (30.77)	
Postural instability – n (%)			0.02
Yes	19 (95.00)	1 (5.00)	
No	8 (61.54)	5 (38.46)	
Gait freezing – n (%)			0.14
Yes	2 (50.00)	2 (50.00)	
No	25 (86.21)	4 (13.79)	
RBD – n (%)			1
Yes	11 (84.62)	2 (15.38)	
No	16 (80.00)	4 (20.00)	
Early gait ataxia (within 3 years of disease onset) – n (%)			1
Yes	1 (100.00)	0 (0.00)	
No	26 (81.25)	6 (18.75)	
Gait ataxia – n (%)			1
Yes	5 (83.33)	1 (16.67)	
No	22 (81.48)	5 (18.52)	
Limb ataxia – n (%)			0.56
Yes	5 (100.00)	0 (0.00)	
No	22 (78.57)	6 (21.43)	
Cerebellar dysartria – n (%)			0.56
Yes	6 (100.00)	0 (0.00)	
No	21 (77.78)	6 (22.22)	
Hyposmia – n (%)			1
Yes	4 (80.00)	1 (20.00)	
No	23 (82.14)	5 (17.86)	
Autonomic failure – n (%)			0.62
Yes	21 (84.00)	4 (16.00)	
No	6 (75.00)	2 (25.00)	
Orthostatic hypotension – n (%)			1
Yes	14 (82.35)	3 (17.65)	
No	13 (86.67)	2 (13.33)	
Other cardiovascular dysautonomic symptoms – n (%)			1
Yes	20 (83.33)	4 (16.67)	
No	7 (77.78)	2 (22.22)	
Urinary disfunction – n (%)			0.29
Yes	23 (85.19)	4 (14.81)	
No	4 (66.67)	2 (33.33)	
Other dysautonomia – n (%)			0.14
Yes	22 (88.00)	3 (12.00)	
No	5 (62.50)	3 (37.50)	
MRI atrophy – n (%)			1
Yes	9 (81.82)	2 (18.18)	
No	17 (80.95)	4 (19.05)	
Age at brushing – mean (SD)	61.22 (7.88)	61.50 (7.82)	0.94
Disease duration at sampling (year) – mean (SD)	5.37 (3.55)	7.83 (6.24)	0.19
Beneficial L-DOPA response UPDRS – mean (SD)	19.52 (10.76)	21.67 (11.06)	0.66
MDS-UPDRS-III OFF – mean (SD)	34.48 (8.87)	36.33 (12.40)	0.67

*SD* standard deviation

*p-values from fisher exact test or t-test, as appropriate

### The biochemical properties of αSyn_RT-QuIC end products enabled an efficient discrimination between α-synucleinopathies

**Fig. 2:**
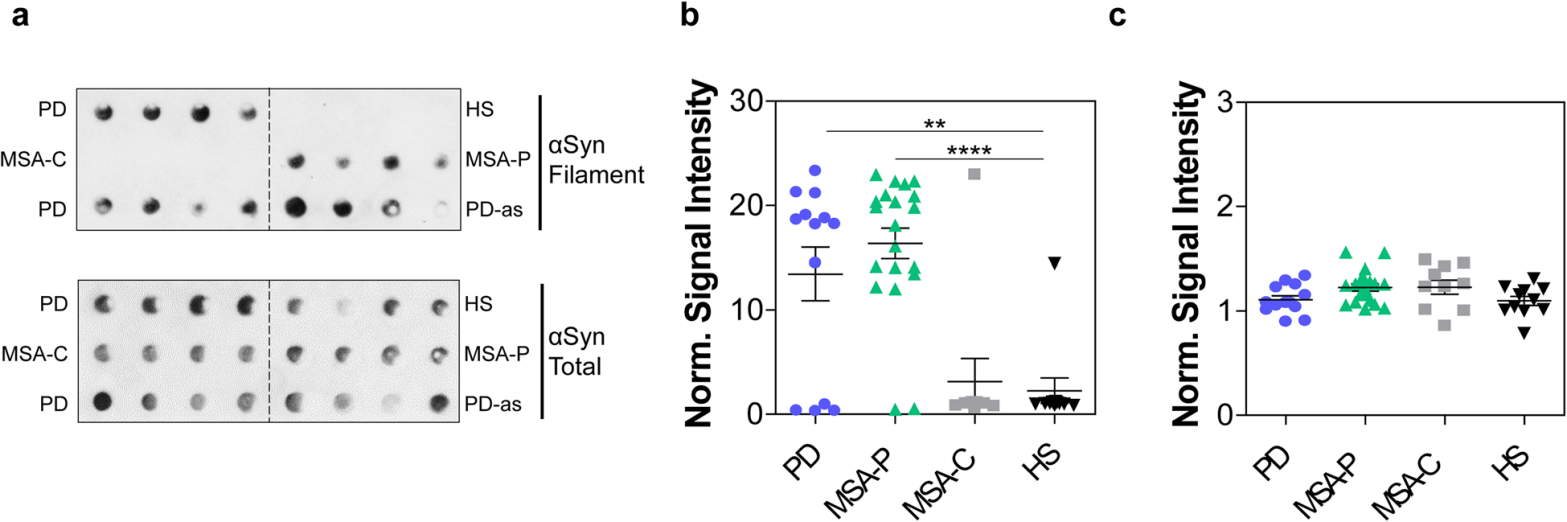
Dot blot immunoassay of αSyn_RT-QuIC products from OM-seeded reactions. Representative dot blot images of αSyn_RT-QuIC end products from OM samples of patients with PD (*n* = 2), MSA-P (*n* = 1), MSA-C (*n* = 1), and from HS (*n* = 1) obtained using an antibody against αSyn filament or antibody against total αSyn (**a**). The αSyn_RT-QuIC product of an autopsy skin sample from a PD case (PD-as) was used as a positive control [18]. The figure shows that αSyn filament immunoreactivity was present only in the products generated by OM samples of PD and MSA-P but was instead absent in those produced by MSA-C and HS. The total αSyn immunoreactivity was observed in all OM samples. Statistical analysis revealed significantly higher levels of αSyn filament in αSyn_RT-QuIC products generated by OM from PD (*p* < 0.01) and MSA-P (*p* < 0.0001) compared to those of MSA-C and HS (one-way ANOVA) (**b**). Total αSyn levels were comparable between groups (*p* = 0.0619, one-way ANOVA) (**c**). The signal intensity of each dot was normalized to that of HS controls (set to the unit of 1) and represented as mean with SD in (**b**) and (**c**). Densitometric analyses in (**b**) and (**c**) have been performed on dot blot images of αSyn_RT-QuIC products generated from PD (*n* = 13), MSA-P (*n* = 20), MSA-C (*n* = 10) and HS (*n* = 11) OM samples at USA-lab (see Supplementary Fig. 3b)

The αSyn_RT-QuIC products seeded with OM samples of PD, MSA-P, MSA-C, and HS were subjected to dot blot immunoassay with a conformation-specific antibody against αSyn filament and revealed the presence of filament-specific αSyn only in samples that induced rec-αSyn aggregation (Fig. 2a,b and Supplementary Fig. 3b). No significant differences were observed in the total levels of αSyn among different groups (Fig. 2a,c and Supplementary Fig. 3b). Therefore, the seeding activity of αSyn_RT-QuIC reactions induced by OM samples from PD and MSA-P can be accounted for by the newly formed αSyn fibrils. In contrast, the lack of seeding activity in OM samples from MSA-C and HS is consistent with the absence of detectable αSyn fibrils following αSyn_RT-QuIC reactions.

**Fig. 3:**
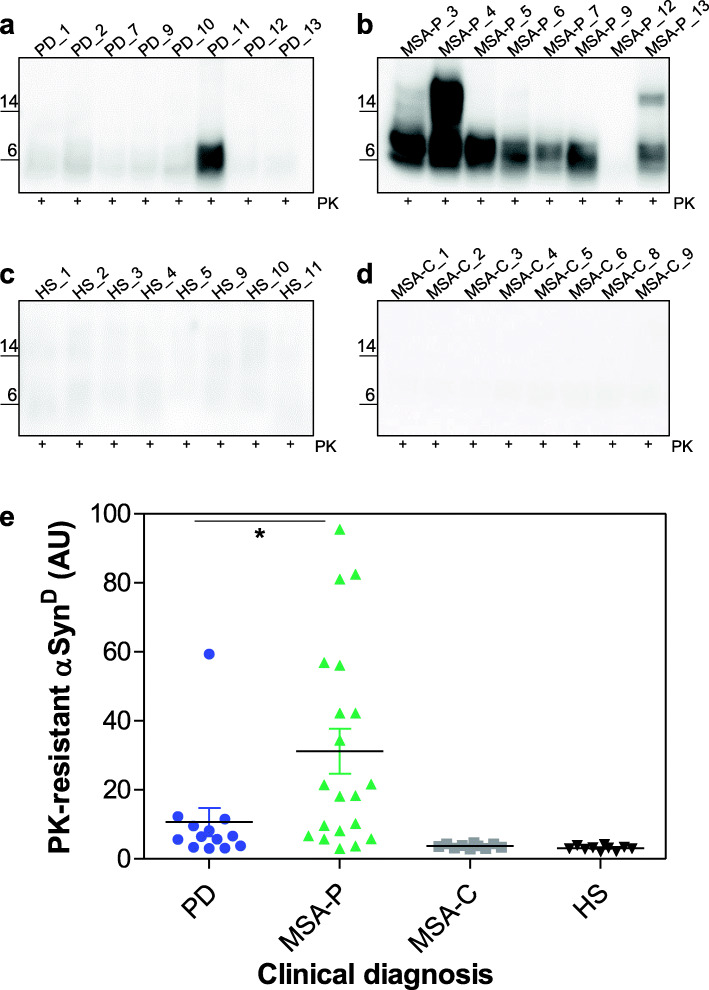
Western blot analyses of αSyn_RT-QuIC products from OM-seeded reactions. Representative Western blot images showing the presence of PK resistant bands in αSyn_RT-QuIC end products generated by PD (*n* = 8) (**a**) and MSA-P (*n* = 8) samples (**b**). Conversely, no bands were detected in αSyn_RT-QuIC end products seeded with HS (*n* = 8) (**c**) and MSA-C (*n* = 8) (**d**). Statistical analysis revealed that PD generated products were significantly less resistant to PK digestion compared to those generated by MSA-P (Mann-Whitney test *p* = 0.0104). Densitometric analyses have been performed on Western blot images of αSyn_RT-QuIC products generated from PD (*n* = 13), MSA-P (*n* = 20), MSA-C (*n* = 10) and HS (*n* = 11) OM samples at ITA-lab (see Supplementary Fig. 3a). Median with interquartile range is shown (**e**)

The same OM-seeded αSyn_RT-QuIC products were then subjected to Western blot analysis after PK treatment and showed the presence of PK-resistant bands in 14/18 MSA-P (78%) and 6/9 (67%) PD samples that induced rec-αSyn aggregation (Fig. 3a,b and Supplementary Fig. 3a). Notably, the bands observed in MSA-P were significantly more resistant to digestion than those observed in PD (Mann-Whitney test, *p* = 0.0104) (Fig. 3e). None of the samples belonging to MSA-C or HS showed PK resistant signals (Fig. 3c,d and Supplementary Fig. 3a).

This difference is probably due to the likelihood that the rec-αSyn substrate was able to acquire distinct abnormal conformations which partially recapitulated those of the original αSyn^D^ strains responsible for PD and MSA-P. This may result in important differences in the PK resistance of the αSyn_RT-QuIC reaction products generated from OM samples of PD and MSA-P. Finally, the αSyn_RT-QuIC products obtained from the analysis of MSA-P and MSA-C samples at ITA-lab (collected at 13 h) were analyzed by Transmission Electron Microscopy (TEM) and confirmed the presence of amyloid fibrils only in MSA-P samples capable of inducing an efficient seeding activity (except for MSA-P_10 and MSA-P_11 that remained negative by RT-QuIC). The amyloid fibrils were not detected in all MSA-C seeded samples (Supplementary Fig. 4). Similarly, when tested with different fluorescent probes known to bind protein aggregates (dye-binding assay), including ThT, Congo red and amyloid-selective marker Amytracker 630 [24], 18 out of 20 MSA-P seeded samples showed a strong fluorescence signal that was instead very weak or absent in all the other MSA tested samples (MSA-P_10, MSA-P_11 and all MSA-C) (Supplementary Fig. 5a). Finally, the epitope mapping study performed on Western blot showed the presence of PK resistant rec-αSyn only in MSA-P samples, thus further supporting these findings (Supplementary Fig. 5b). Combined together, these results confirmed that there was a good correlation between the αSyn_RT-QuIC seeding activity and the presence of amyloid fibrils in each sample, and that, in contrast to MSA-P, the MSA-C samples did not efficiently trigger rec-αSyn aggregation.

Therefore, thanks to the combination of these biochemical and morphological analyses, we were able to validate the αSyn_RT-QuIC results, recognize PD and MSA-P, and even discriminate MSA-P from MSA-C.

## Discussion

Accurate diagnoses of PD and MSA early on in their clinical course is challenging because symptoms often overlap [25]. Misdiagnosis negatively impacts the healthcare system and the life of the patients and could lead to erroneous inclusion in clinical trials. Proper diagnosis can be predictive of treatment responsiveness, indeed, in the early stage, MSA-P patients often respond to levodopa while MSA-C patients do not [26]. It is, therefore, necessary to identify the disease phenotype as early as possible, to provide MSA patients with the most appropriate diagnostic and therapeutic care pathways. The same concept is valid for the other α-synucleinopathies, including PD, where early and accurate diagnosis allows proper treatment initiation and is key to enrollment into neuroprotective clinical trials. With the development of the seeding aggregation assays (SAAs), including RT-QuIC, significant progress has been made in the field of α-synucleinopathy diagnosis. For instance, through RT-QuIC analysis, traces of αSyn^D^ were found in the CSF, OM, submandibular gland, and, very recently, skin of diseased patients [12–19]. The identification of αSyn^D^ in peripheral tissues may serve as the gold standard for the diagnosis of α-synucleinopathies. For this reason, once optimized and integrated into clinical practice, SAAs have the potential to revolutionize the diagnosis, therapy, and prognostication of these diseases.

The majority of the RT-QuIC studies applied to α-synucleinopathies were based on CSF samples and highlighted that, besides the impressive performance of the technology in detecting traces of αSyn^D^, there are important variabilities in the findings that could be associated with the experimental protocol adopted by each laboratory. The use of customized analytical procedures has hampered the possibility to compare the results and evaluate the reliability and the robustness of the assays. For this reason, we have combined for the first time the expertise of two independent specialized laboratories (ITA-lab and USA-lab) with the aim to explore the interlaboratory reproducibility of RT-QuIC performed on OM samples collected from PD, MSA-C, and MSA-P patients. In contrast to CSF collection that can be painful and associated with adverse effects such as headache or cerebrospinal fluid leak, OM samples can be easily and periodically collected with a minimally invasive procedure. Both of our laboratories harmonized the analytical procedures by minimizing all the experimental variables that could lead to inconsistencies in the results or conflicting findings (e.g. use of a commercially available rec-αSyn). The final αSyn_RT-QuIC protocol that has been developed enabled us to reach a very high IAR (96%) between ITA-lab and USA-lab. In particular, the OM of 69% PD patients and 90% MSA-P patients induced αSyn_RT-QuIC seeding activity with 100% of IAR. In contrast, none of the OM belonging to MSA-C patients and the HS induced αSyn_RT-QuIC seeding activity at ITA-lab, except for one patient per group at USA-lab, thus leading to 90 and 91% IAR, respectively. We decided to consider non age-matched HS (younger than the patients) for reducing the probability to include subjects at pre-symptomatic stages of α-synucleinopathy, eventually causing positive αSyn_RT-QuIC results difficult to interpret (e.g. real seeding activity vs false positive signal). Surprisingly, OM collected from MSA-P and MSA-C patients showed totally different behavior, with the latter being almost unresponsive to αSyn_RT-QuIC. The lack of seeding activity in MSA-C suggests that, although MSA-P and MSA-C belong to the same disease group, they may be caused by distinct αSyn^D^ strains that possess different tropism for peripheral tissues, including the OM. In very recent studies, it has been suggested that αSyn^D^ may originate outside the brain, including the nose, the gut and the urogenital tract and then spread to the CNS [27–29]. The tissue microenvironment might influence αSyn^D^ properties thus driving its tropism for specific neuroanatomical regions. This might lead to the onset of different forms of MSA and contribute to the phenotypic heterogeneity of α-synucleinopathies.

Interesting findings indicate that even different αSyn strains can be found within the same MSA brain [30]. Since the brain homogenates of MSA-C patients were able to induce an efficient αSyn_RT-QuIC seeding activity using our protocol, we speculate that the αSyn^D^ strain responsible for MSA-C (i) might not be present in the OM (while that associated with MSA-P accumulates in this tissue with greater efficiency), (ii) might be present in the OM but could be subjected to tissue/microenvironment specific changes that make this strain incapable of triggering the seeding activity, or (iii) might be present in the OM but at concentrations which are still too low to induce a detectable seeding activity. Regardless of the reasons which determine this opposite αSyn_RT-QuIC behavior of MSA-P and MSA-C, the findings can be exploited to efficiently distinguish these different pathologies in living patients. Several research groups have subjected to RT-QuIC analysis the CSF of patients with MSA but controversial results were reported [15, 31–34]. Moreover, except for a few papers, there were no clear indications about the subtypes of MSA examined and whether specific correlations between the disease phenotype and the RT-QuIC outcomes were observed. Interestingly, van Rumund and colleagues reported that only a small group of CSF samples belonging to MSA-P patients were able to induce seeding activity by RT-QuIC, while those belonging to MSA-C patients did not [33]. Although these data refer to a different biological sample, they are in line with those of our OM study and further support the hypothesis that MSA-P and MSA-C may be caused by distinct αSyn^D^ strains with peculiar tropism for CSF, OM, and likely other peripheral tissues. Our findings might also unveil different biological and molecular pathways involved in the disease pathogenesis.

Notably, in PD and MSA-P patients we found a positive significant association between αSyn_RT-QuIC seeding activity and some clinical parameters, including rigidity and postural instability. In contrast, we did not find any correlation between αSyn_RT-QuIC seeding activity and the disease duration.

Taken together, these data indicate that through a careful combination of aggregation kinetics, biochemical and morphological assays, and clinical information, the αSyn_RT-QuIC assay of OM can significantly improve the clinical diagnosis of α-synucleinopathies. In particular, the assay may help physicians to identify and stratify patients with PD and MSA and, above all, to specifically recognize MSA-P or MSA-C phenotypes.

The high degree of interlaboratory reproducibility strongly supports the reliability and the robustness of the αSyn_RT-QuIC results. Therefore, the fact that some OM of PD patients and almost all OM of MSA-C patients did not induce αSyn_RT-QuIC seeding activity does not represent a technical limitation of the assay (e.g. lack of sensitivity). These findings could also not be the result of stochastic events. We rather think that there might be additional significant biological and pathophysiological reasons that cannot be totally understood at present. Certainly, we can benefit from better-recognizing patients with PD, MSA-P, and MSA-C, and potentially identifying heterogeneous pathological subgroups within these diagnoses, in order to pave the way to tailored treatment regimens.

For all these reasons, OM-based αSyn_RT-QuIC analysis may be a promising candidate as a routine diagnostic test for PD, MSA-P, and MSA-C. Additionally, this assay requires further studies to determine whether it will identify presymptomatic and prodromal PD and MSA patients. If this were the case, αSyn_RT-QuIC could be helpful to identify subjects at risk of developing the disease, and can be particularly important in this very moment where preliminary studies suggest that the Sars-CoV-2 viral infection seems to influence the vulnerability to PD [35]. Finally, the assay can be exploited to test in vitro the efficacy of therapeutic compounds to interfere with the aggregation of αSyn in reactions seeded with patients’ biological samples.

The fact that few, if any, of the patients included in our work will undergo neuropathological assessment, represents the major limitation of the study that cannot be addressed. The lack of OM samples collected from pathologically confirmed cases of PD, MSA-C, and MSA-P hampers the possibility to perform a retrospective analysis for estimating the sensitivity and specificity of the assay. Moreover, the quantity of OM sample collectible from each patient is considerably limited. For this reason, we have decided to perform this preliminary work of protocol set-up and harmonization by involving no more than two specialized laboratories with the aim of having enough material to be accurately and repeatedly analyzed by both groups. As a matter of fact, almost the entire volume of each OM sample included in this work was consumed during the analyses before reaching the conclusion that the samples can be significantly diluted (up to 20X) without affecting the sensitivity and specificity of the αSyn_RT-QuIC. This observation should be taken into account for future studies, where newly collected OM can be prepared as described and used to perform multicenter studies for a better assessment of the αSyn_RT-QuIC performances. The limited number of samples included in this study depends on the fact that MSA is a rare disease and that the COVID-19 pandemic outbreak has imposed severe limitations on OM collection for biosafety reasons. Nevertheless, our exploratory study showed striking differences in αSyn_RT-QuIC responses between (1) MSA-C and MSA-P, (2) MSA-C and PD, (3) MSA-P and controls, and (4) PD and controls that were faithfully reproducible in both laboratories thus demonstrating that they were not due to merely stochastic events. Although the analytical procedures have been harmonized, two different thresholds of time were set at ITA-lab and USA-lab to discriminate positive and negative samples. Likely, despite using the same instrument (BMG LabTech OMEGA), some conditions may inevitably differ (e.g. the stability of the temperature during the whole analytical process or the mechanism of shaking of the plate) hence promoting the variability in the time thresholds between labs. In addition, other factors, including the precision of the pipettes or the type of consumables (e.g. tubes) used for the analysis might have favored this discrepancy. Certainly, additional studies would help to define a window of time, instead of a single lab-dependent time-threshold, within which the samples can be considered positive.

Longitudinal studies using samples collected from larger cohorts of patients are required to definitively evaluate the diagnostic accuracy of OM-αSyn^D^ as a biomarker for PD, MSA-P, MSA-C, and other α-synucleinopathies. However, we believe that these evaluations should be performed at the end of the COVID-19 pandemic since, at present, we do not know whether the potential presence of the Sars-CoV-2 virus in the nasal cavity of either diseased patients or healthy subjects might alter the properties of the OM samples while compromising the αSyn_RT-QuIC analyses.

Finally, additional investigations will also be needed to determine whether this assay could be used as a biomarker to track disease progression and monitor the effect of disease-modifying treatments.

## Conclusions

The use of the ultrasensitive RT-QuIC assay could potentially help the field of α-synucleinopathy diagnosis, but a process of assay harmonization is urgently needed to minimize the variability or conflicting findings between specialized laboratories. Our results suggest that PD, MSA-P, and MSA-C are caused by distinct αSyn^D^ strains that might have peculiar pathological features and tropism for peripheral tissues, including OM, eventually unveiling different biological and molecular pathways involved in the disease pathogenesis. This study provides evidence that αSyn_RT-QuIC of OM samples represents a reliable assay that can distinguish patients with MSA-P from those with MSA-C, and may limit the negative effects that misdiagnosis produces in terms of costs for the healthcare system and improve overall patient care, treatment, and possible enrollment in future clinical trials.

## Supplementary Information

**Additional file 1. **Detection of αSyn_RT-QuIC seeding activity triggered by the brain homogenates of patients with PD, MSA-P, and MSA-C. We tested the performance of the new αSyn_RT-QuIC protocol using the brain homogenates (BH) of autopsy-confirmed cases of PD (*n* = 1), MSA-P (*n* = 1), and MSA-C (*n* = 1) as sources of different αSyn^D^ strains. The BH of a patient not affected by α-synucleinopathy (NAS) was used as control. Immunohistochemical analysis revealed the presence of distinct αSyn^D^ aggregates in the brain of tested patients, except for NAS. MSA-P and NAS images were taken using 10x magnification. MSA-C and PD images were taken using 40x magnification (a). Frozen brain samples (substantia nigra for PD, striatum for MSA-P, cerebellum for MSA-C, and frontal cortex for NAS) were homogenized at 10% in PBS (weight/volume) and diluted at 10^− 12^. One μl of each BH was added to 49 μL of reaction mix and subjected to αSyn_RT-QuIC analysis. The results indicate that PD (light green line), MSA-P (blue line), and MSA-C (grey line) induced αSyn_RT-QuIC seeding activity before the time threshold set at 13 h while that of the patient with NAS (purple line) did not (b). Each sample was analyzed in quadruplicate. Curves represented in the graph were obtained by plotting the average fluorescence intensities of each sample against time.

**Additional file 2. **Time to threshold and mean fluorescence at time threshold obtained at ITA-lab and USA-lab. At ITA-lab, time to threshold was significantly shorter in reactions seeded with OM samples of PD compared to that of MSA-P (unpaired t-test, *p* = 0.017) (a) while at USA-lab they were comparable (unpaired t-test, *p* = 0.3944) (b). No significant differences in the average of fluorescence values reached by PD and MSA samples at the time threshold were observed in both ITA-lab (146,494 ± 103,670 AU (mean ± SD) and 147,298 ± 80,123 AU, respectively; Mann-Whitney test, *p* = 0.9853) (c) and USA-lab (108,961 ± 110,426 AU and 119,929 ± 83,655 AU, respectively; Mann-Whitney test, *p* = 0.4279) (d). In a and b, means with 95% CI are shown. In c and d, medians with interquartile range are shown.

**Additional file 3. **Biochemical analysis of αSyn_RT-QuIC reaction products. Western blot images of αSyn_RT-QuIC reaction products obtained at ITA-lab from OM samples of all individuals (PD = 13, MSA-P = 20, MSA-C = 10 and HS = 11) that were used for quantification analysis reported in Fig. 3e. Samples were digested with PK (2.5 mg/mL) and immunoblotted with the rabbit polyclonal α-synuclein antibody Agrisera AS08 358 (1:1,000). PK resistant bands were observed in several αSyn_RT-QuIC end products seeded with PD and MSA-P samples while they were not detected in all MSA-C and HS seeded samples. Vertical dashed lines in each blot indicate cropped images from separate gels (a). Dot blot images of αSyn_RT-QuIC reaction products obtained at USA-lab from OM samples of all individuals (PD = 13, MSA-P = 20, MSA-C = 10 and HS = 11) that were used for quantification analysis reported in Fig. 2b and c. Total αSyn signal from these products was present in all samples while αSyn filament signal was present only in samples which induced αSyn_RT-QuIC seeding activity (b).

**Additional file 4.** Morphological analsysis of αSyn_RT-QuIC reaction products generated by MSA-P and MSA-C samples at ITA-lab. αSyn_RT-QuIC reaction products were properly diluted and 10 μL of the final dilutions was dropped onto 200-mesh Formvar-carbon coated nickel grids for 30 min and the remaining drop was blotted dry using filter papers. Samples were stained with 25% Uranyl Acetate Replacement (UAR, negative staining) for 10 min, the solution was removed using filter papers and the grids were air-dried for 15 min before the analyses. Images were recorded at 120 kV with a FEI Tecnai Spirit, equipped with an Olimpus Megaview G2 camera. Rec-αSyn amyloid fibrils were detected in all αSyn_RT-QuIC products seeded with MSA-P samples, except for MSA-P_10 and MSA-P_11. In constrast, none of the MSA-C samples induced the formation of rec-αSyn fibrils (except for occasional, rare and short aggregates detected in MSA-C_1, MSA-C_3 and MSA-C_6). All TEM images were taken at the same magnification (scale bar: 200 nm).

**Additional file 5. **Dye-binding assay and epitope mapping of αSyn_RT-QuIC products generated by MSA-P (*n* = 20) and MSA-C (*n* = 10) samples at ITA-lab. Thirty-five μL of αSyn_RT-QuIC products, generated without the use of ThT, were incubated with three different fluorescent dyes: (i) ThT [10 μM], (ii) Congo Red [5 μM] and (iii) Amytracker 630 (purchased from Ebba Biotech and diluted 1:800) for 30 min in the dark at room temperature. Fluorescent signals were measured with the appropriate wave-lengths (448 exc/482 emi for ThT, 544 exc/620 emi for Congo red and 510 exc/635 emi for Amytracker 630) using the BMG LabTech CLARIOSTAR microplate reader. Regardless of the probe used, all MSA-P samples (except for MSA-P_10 and MSA-P_11) showed strong fluorescent signals while the MSA-C did not. These differences were statistically significant in the case of (i) ThT (*t*-test *p* = 0.0063), (ii) Congo red (*t*-test, *p* = 0.0018) and (iii) Amytracker 630 (t-test, *p* = 0.0051). Graphs show the mean values (± SEM) of MSA-P and MSA-C fluorescent signals obtained from (i) ThT (graph on the left), (ii) Congo red (graph in the middle) and (iii) Amytracker 630 (graph on the right) analysis (a). Eight μL of αSyn_RT-QuIC products generated by MSA-P and MSA-C samples were treated with PK [2.5 mg/mL] for 1 h at 37 °C under shaking (500 rpm) and immunoblotted with antibodies directed against three different epitopes of α-synuclein: (i) clone 5C2 (epitopes 61–95, Novus Biologicals), (ii) AB5038 (epitopes 111–131, Chemicon international), and (iii) clone 42 (epitopes 15–123, BD Bioscience). No signals were observed in samples immunoblotted with the AB5038 and the clone 5C2, while with the clone 42, PK resistant bands were specifically detected in αSyn_RT-QuIC products seeded with MSA-P but not in those seeded with MSA-C samples (b).

## Data Availability

All data generate or analysed during this study are included in this published article (and its supplementary information files).

## Abbreviations

αSyn: α-synuclein
MSA: Multiple system atrophy
PD: Parkinson’s disease
OM: Olfactory mucosa
αSyn_RT-QuIC: α-synuclein Real-Time Quaking-Induced Conversion
MSA-P: Multiple system atrophy with the parkinsonian phenotype
MSA-C: Multiple system atrophy with the cerebellar phenotype
HS: Healthy control subjects
IAR: Interrater agreement of results
MRI: Magnetic resonance imaging
RT-QuIC: Real-Time Quaking-Induced Conversion
CSF: Cerebrospinal fluid
MDS-UPDRS: Movement Disorder Society-Unified Parkinson’s Disease Rating Scale
I-123 MIBG: Iodine 123 (^123^I) metaiodobenzylguanidine
SPET-DATSCAN: Single Photon Emission Tomography - Dopamine Transporter Scan
FDG-PET: ^18^F-fluorodeoxyglucose Positron Emission Tomography
RBD: Rapid eye movement sleep Behavior Disorder
L-DOPA: Levodopa
PBS: Phosphate-buffer saline
rec-αSyn: Recombinant human α-synuclein
ThT: Thioflavin-T
AU: Arbitrary units
SD: Standard deviation
SEM: Standard error of the mean
BSA: Bovine serum albumin
TBST: Tris-Buffered Saline containing Tween-20
PK: Proteinase K
PVDF: Polyvinylidene difluoride
BH: Brain homogenate
TEM: Transmission Electron Microscopy
SAA: Seeding aggregation assay
COVID-19: COronaVIrus Disease 19
Sars-CoV-2: Severe Acute Respiratory Syndrome Coronavirus-2
