# Supplementary figures and images for: Discrimination of MSA-P and MSA-C by RT-QuIC analysis of olfactory mucosa: the first assessment of assay reproducibility between two specialized laboratories
Source: Mol Neurodegener. 2021 Dec 11;16:82. doi: 10.1186/s13024-021-00491-y (PMC8665327; doi:10.1186/s13024-021-00491-y)

**ADDITIONAL FILE 1**

**
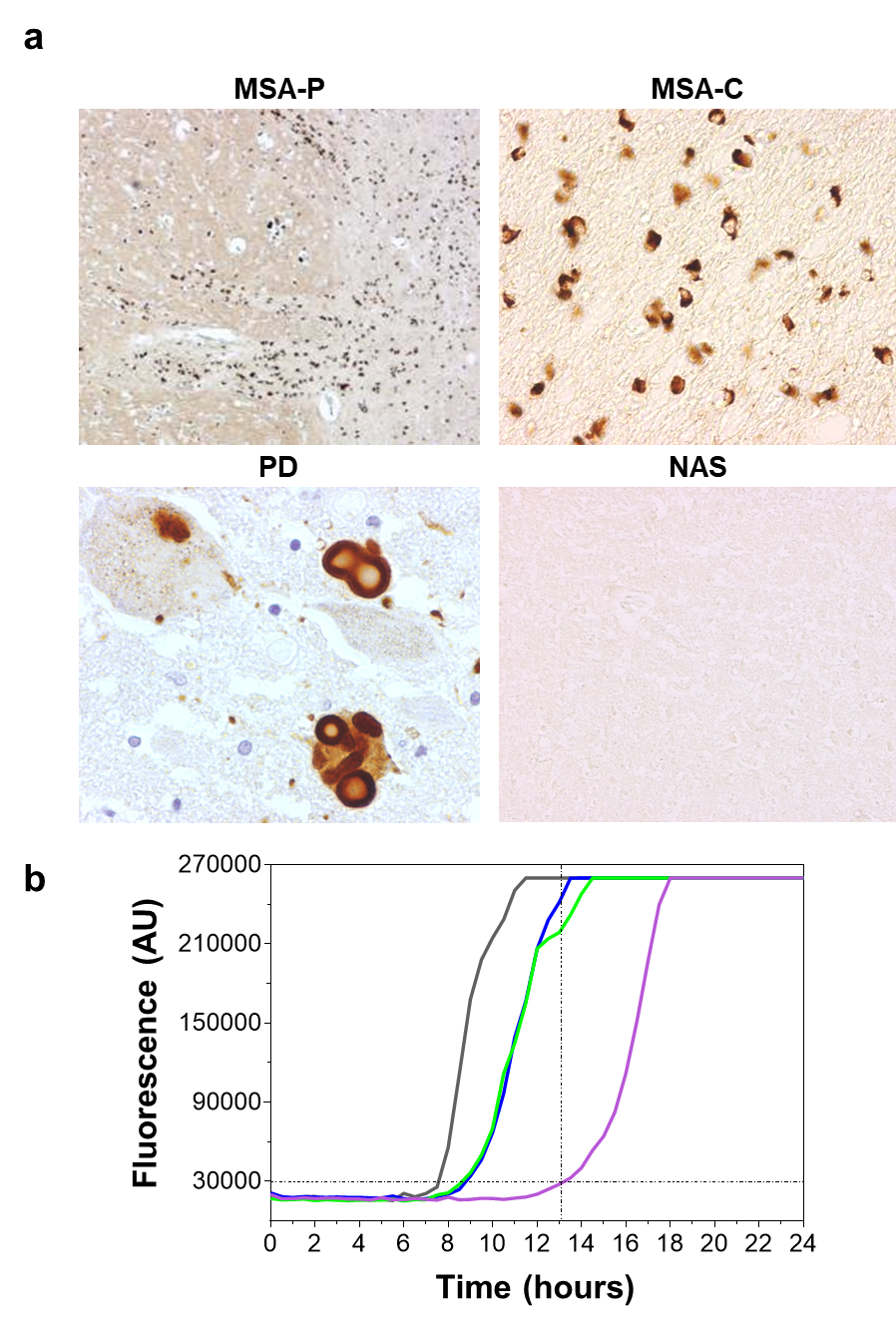
**

Supplement: Supplementary file 1 — Additional file 1. Detection of αSyn_RT-QuIC seeding activity triggered by the brain homogenates of patients with PD, MSA-P, and MSA-C. We tested the performance of the new αSyn_RT-QuIC protocol using the brain homogenates (BH) of autopsy-confirmed cases of PD (n = 1), MSA-P (n = 1), and MSA-C (n = 1) as sources of different αSynD strains. The BH of a patient not affected by α-synucleinopathy (NAS) was used as control. Immunohistochemical analysis revealed the presence of distinct αSynD aggregates in the brain of tested patients, except for NAS. MSA-P and NAS images were taken using 10x magnification. MSA-C and PD images were taken using 40x magnification (a). Frozen brain samples (substantia nigra for PD, striatum for MSA-P, cerebellum for MSA-C, and frontal cortex for NAS) were homogenized at 10% in PBS (weight/volume) and diluted at 10− 12. One μl of each BH was added to 49 μL of reaction mix and subjected to αSyn_RT-QuIC analysis. The results indicate that PD (light green line), MSA-P (blue line), and MSA-C (grey line) induced αSyn_RT-QuIC seeding activity before the time threshold set at 13 h while that of the patient with NAS (purple line) did not (b). Each sample was analyzed in quadruplicate. Curves represented in the graph were obtained by plotting the average fluorescence intensities of each sample against time. [file 13024_2021_491_MOESM1_ESM.doc]

**ADDITIONAL FILE 2**

**
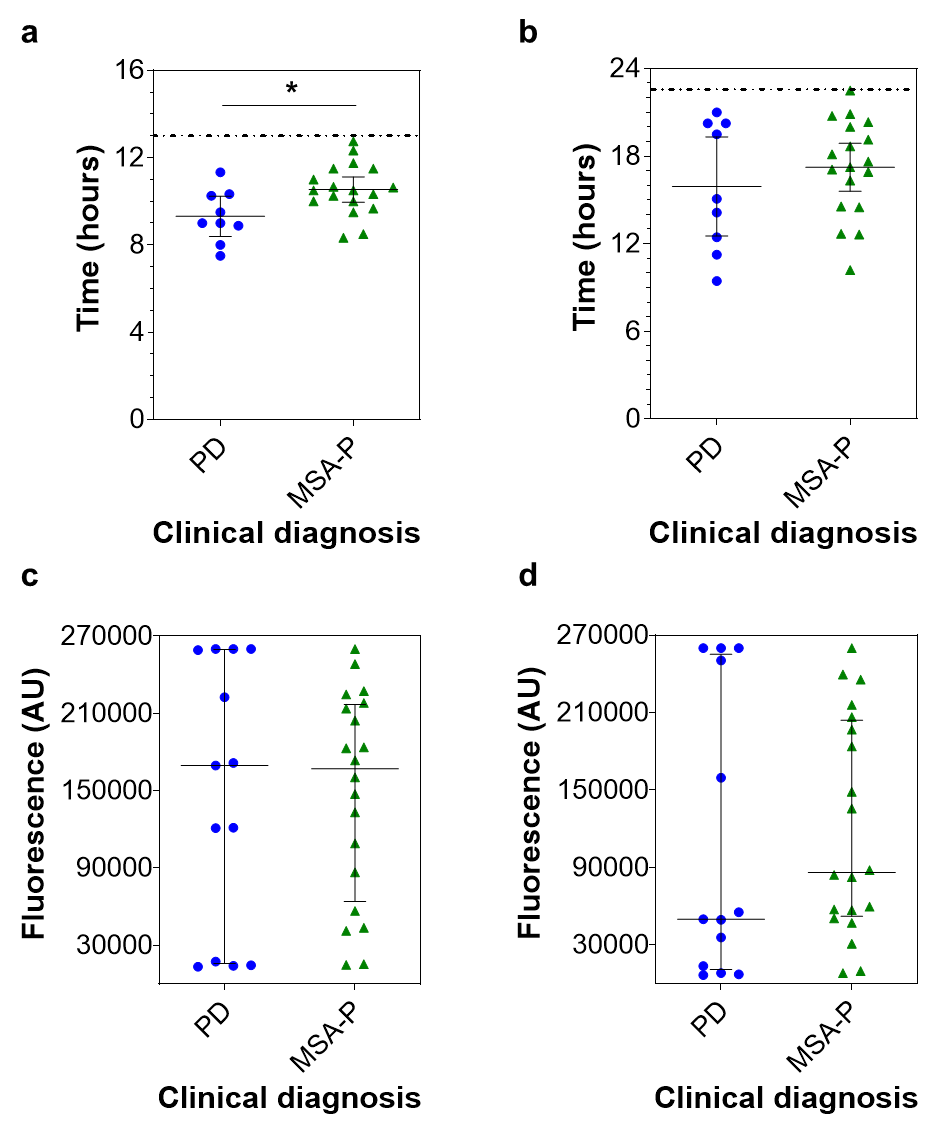
**

Supplement: Supplementary file 2 — Additional file 2. Time to threshold and mean fluorescence at time threshold obtained at ITA-lab and USA-lab. At ITA-lab, time to threshold was significantly shorter in reactions seeded with OM samples of PD compared to that of MSA-P (unpaired t-test, p = 0.017) (a) while at USA-lab they were comparable (unpaired t-test, p = 0.3944) (b). No significant differences in the average of fluorescence values reached by PD and MSA samples at the time threshold were observed in both ITA-lab (146,494 ± 103,670 AU (mean ± SD) and 147,298 ± 80,123 AU, respectively; Mann-Whitney test, p = 0.9853) (c) and USA-lab (108,961 ± 110,426 AU and 119,929 ± 83,655 AU, respectively; Mann-Whitney test, p = 0.4279) (d). In a and b, means with 95% CI are shown. In c and d, medians with interquartile range are shown. [file 13024_2021_491_MOESM2_ESM.doc]

**ADDITIONAL FILE 3**

**
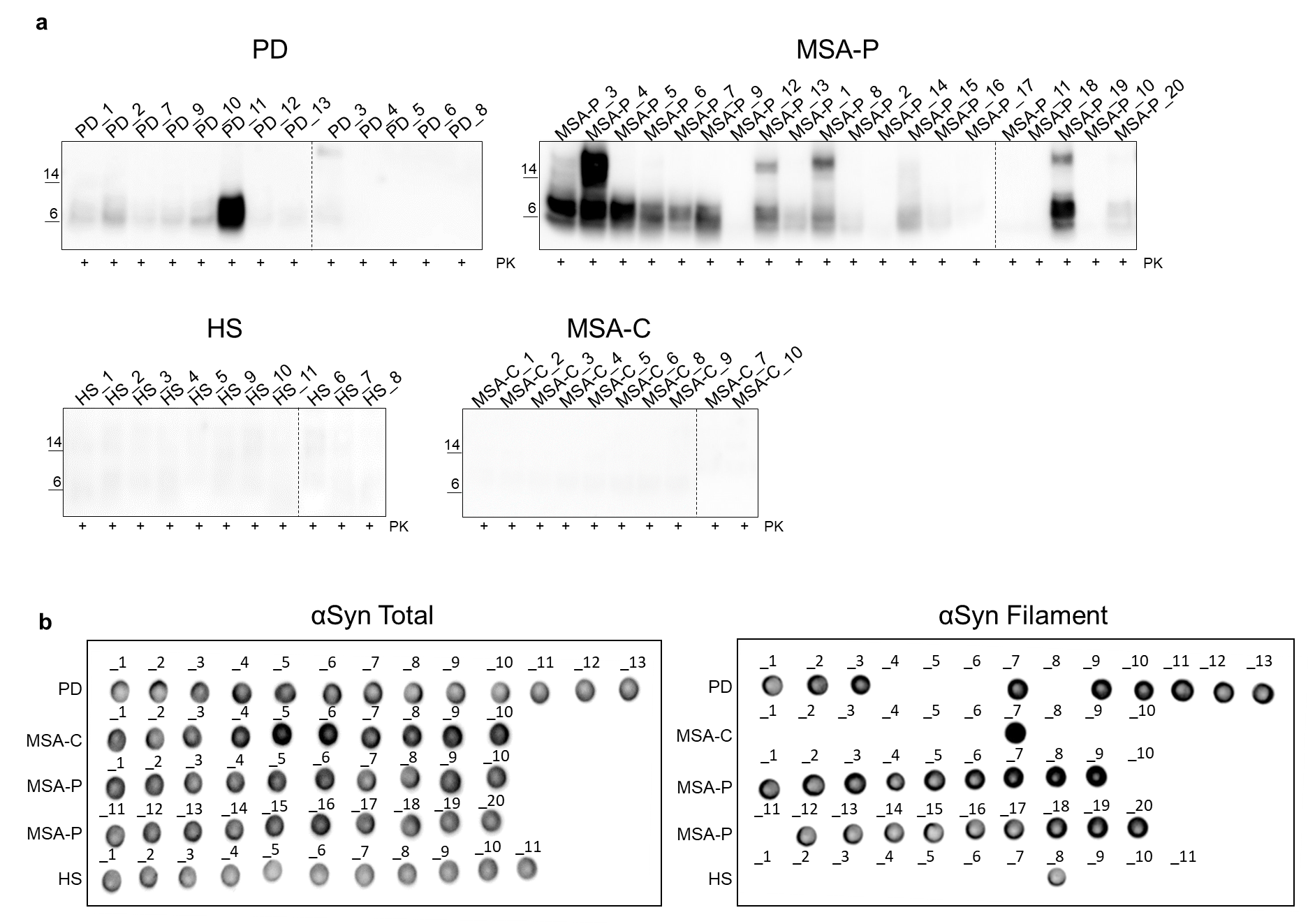
**

Supplement: Supplementary file 3 — Additional file 3. Biochemical analysis of αSyn_RT-QuIC reaction products. Western blot images of αSyn_RT-QuIC reaction products obtained at ITA-lab from OM samples of all individuals (PD = 13, MSA-P = 20, MSA-C = 10 and HS = 11) that were used for quantification analysis reported in Fig. 3e. Samples were digested with PK (2.5 mg/mL) and immunoblotted with the rabbit polyclonal α-synuclein antibody Agrisera AS08 358 (1:1,000). PK resistant bands were observed in several αSyn_RT-QuIC end products seeded with PD and MSA-P samples while they were not detected in all MSA-C and HS seeded samples. Vertical dashed lines in each blot indicate cropped images from separate gels (a). Dot blot images of αSyn_RT-QuIC reaction products obtained at USA-lab from OM samples of all individuals (PD = 13, MSA-P = 20, MSA-C = 10 and HS = 11) that were used for quantification analysis reported in Fig. 2b and c. Total αSyn signal from these products was present in all samples while αSyn filament signal was present only in samples which induced αSyn_RT-QuIC seeding activity (b). [file 13024_2021_491_MOESM3_ESM.doc]

**ADDITIONAL FILE 5**

**
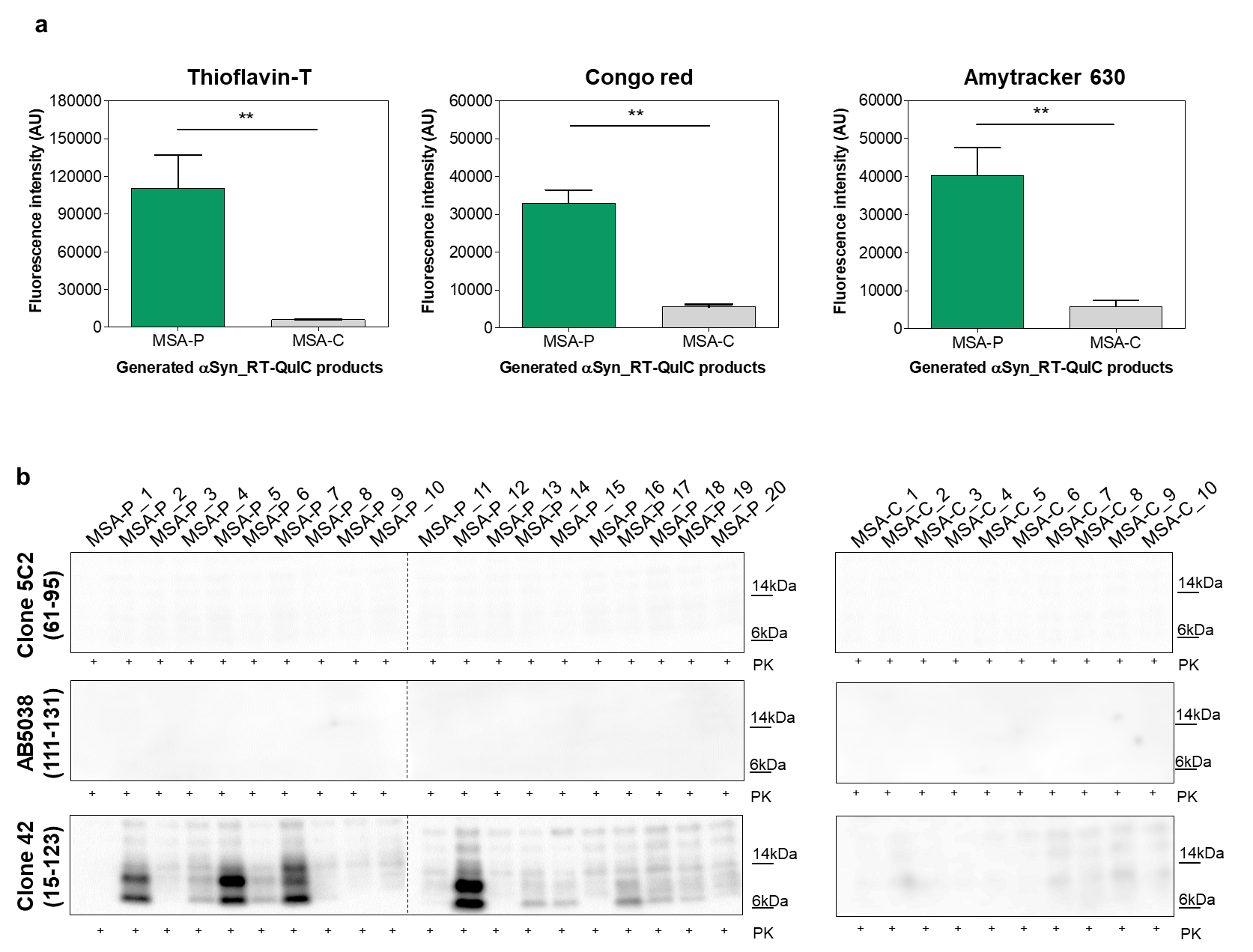
**

Supplement: Supplementary file 5 — Additional file 5. Dye-binding assay and epitope mapping of αSyn_RT-QuIC products generated by MSA-P (n = 20) and MSA-C (n = 10) samples at ITA-lab. Thirty-five μL of αSyn_RT-QuIC products, generated without the use of ThT, were incubated with three different fluorescent dyes: (i) ThT [10 μM], (ii) Congo Red [5 μM] and (iii) Amytracker 630 (purchased from Ebba Biotech and diluted 1:800) for 30 min in the dark at room temperature. Fluorescent signals were measured with the appropriate wave-lengths (448 exc/482 emi for ThT, 544 exc/620 emi for Congo red and 510 exc/635 emi for Amytracker 630) using the BMG LabTech CLARIOSTAR microplate reader. Regardless of the probe used, all MSA-P samples (except for MSA-P_10 and MSA-P_11) showed strong fluorescent signals while the MSA-C did not. These differences were statistically significant in the case of (i) ThT (t-test p = 0.0063), (ii) Congo red (t-test, p = 0.0018) and (iii) Amytracker 630 (t-test, p = 0.0051). Graphs show the mean values (± SEM) of MSA-P and MSA-C fluorescent signals obtained from (i) ThT (graph on the left), (ii) Congo red (graph in the middle) and (iii) Amytracker 630 (graph on the right) analysis (a). Eight μL of αSyn_RT-QuIC products generated by MSA-P and MSA-C samples were treated with PK [2.5 mg/mL] for 1 h at 37 °C under shaking (500 rpm) and immunoblotted with antibodies directed against three different epitopes of α-synuclein: (i) clone 5C2 (epitopes 61–95, Novus Biologicals), (ii) AB5038 (epitopes 111–131, Chemicon international), and (iii) clone 42 (epitopes 15–123, BD Bioscience). No signals were observed in samples immunoblotted with the AB5038 and the clone 5C2, while with the clone 42, PK resistant bands were specifically detected in αSyn_RT-QuIC products seeded with MSA-P but not in those seeded with MSA-C samples (b). [file 13024_2021_491_MOESM5_ESM.doc]
