# Supplementary material for: Discrimination of MSA-P and MSA-C by RT-QuIC analysis of olfactory mucosa: the first assessment of assay reproducibility between two specialized laboratories
Source: Mol Neurodegener. 2021 Dec 11;16:82. doi: 10.1186/s13024-021-00491-y (PMC8665327; doi:10.1186/s13024-021-00491-y)
Supplement: Supplementary file 4 — Additional file 4. Morphological analsysis of αSyn_RT-QuIC reaction products generated by MSA-P and MSA-C samples at ITA-lab. αSyn_RT-QuIC reaction products were properly diluted and 10 μL of the final dilutions was dropped onto 200-mesh Formvar-carbon coated nickel grids for 30 min and the remaining drop was blotted dry using filter papers. Samples were stained with 25% Uranyl Acetate Replacement (UAR, negative staining) for 10 min, the solution was removed using filter papers and the grids were air-dried for 15 min before the analyses. Images were recorded at 120 kV with a FEI Tecnai Spirit, equipped with an Olimpus Megaview G2 camera. Rec-αSyn amyloid fibrils were detected in all αSyn_RT-QuIC products seeded with MSA-P samples, except for MSA-P_10 and MSA-P_11. In constrast, none of the MSA-C samples induced the formation of rec-αSyn fibrils (except for occasional, rare and short aggregates detected in MSA-C_1, MSA-C_3 and MSA-C_6). All TEM images were taken at the same magnification (scale bar: 200 nm). [file 13024_2021_491_MOESM4_ESM.pptx]

## Slide 1
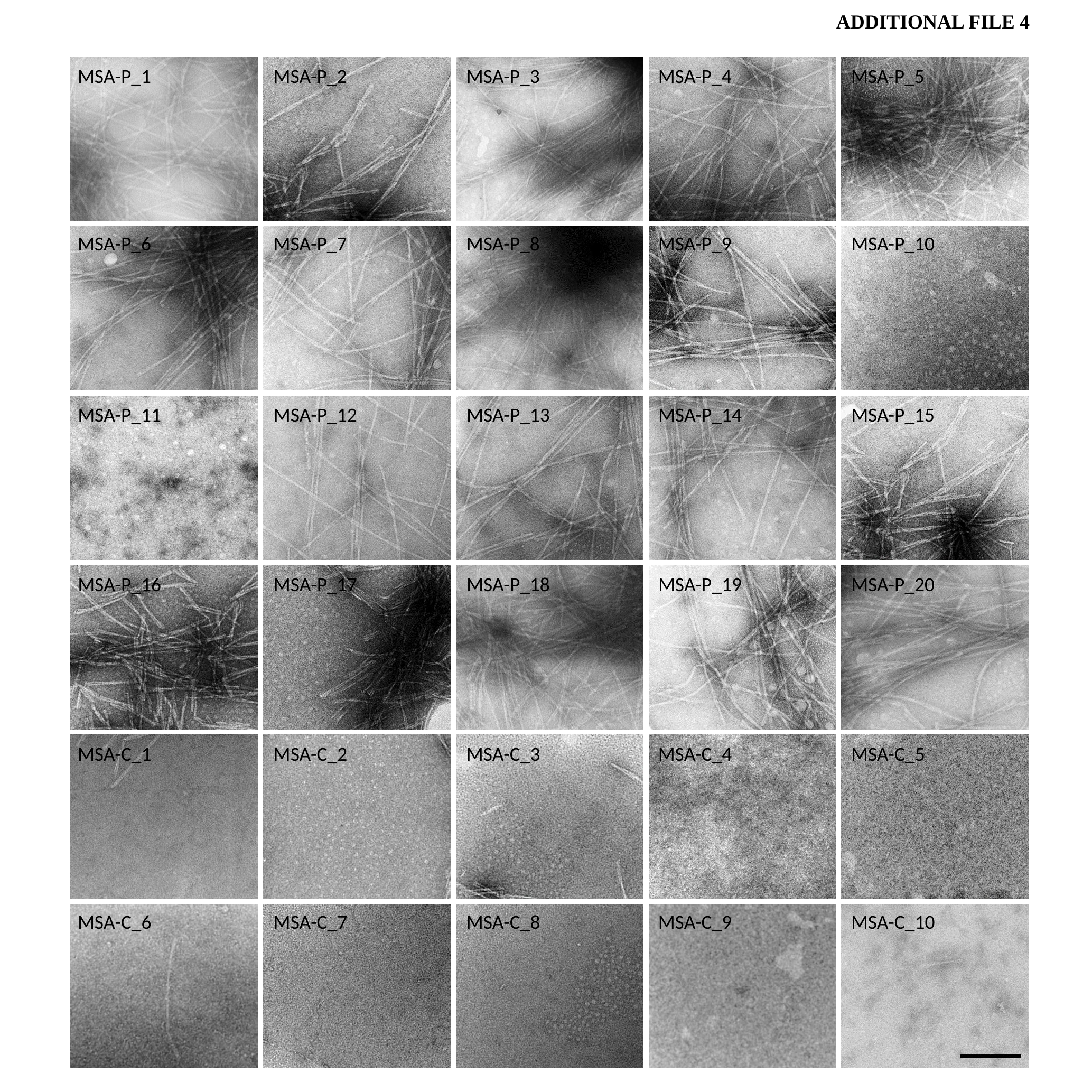

ADDITIONAL FILE 4
MSA-P_1
MSA-P_2
MSA-P_3
MSA-P_4
MSA-P_5
MSA-P_6
MSA-P_7
MSA-P_8
MSA-P_9
MSA-P_10
MSA-P_11
MSA-P_12
MSA-P_13
MSA-P_14
MSA-P_15
MSA-P_16
MSA-P_17
MSA-P_18
MSA-P_19
MSA-P_20
MSA-C_1
MSA-C_2
MSA-C_3
MSA-C_4
MSA-C_5
MSA-C_6
MSA-C_7
MSA-C_8
MSA-C_9
MSA-C_10
